# Selective enrichment of mycobacterial proteins from infected host macrophages

**DOI:** 10.1038/srep13430

**Published:** 2015-08-25

**Authors:** Ajit G. Chande, Zaved Siddiqui, Mukul Kumar Midha, Varsha Sirohi, Srikanth Ravichandran, Kanury V. S. Rao

**Affiliations:** 1 Immunology Group, International Centre for Genetic Engineering and Biotechnology, New Delhi-India; 2 Drug Discovery Research Centre, Translational Health Science and Technology Institute, Gurgaon, Haryana-India

## Abstract

Upon infection, Mycobacterium tuberculosis (Mtb) deploys specialized secretion machinery to deliver virulent proteins with the capacity to modulate a variety of host-cellular pathways. Studies on the identification of intra-macrophage Mtb proteins, however, are constricted by an inability to selectively enrich these virulent effectors against overwhelming protein content of the host. Here, we introduce an Mtb-selective protein labeling method based on genetic incorporation of azidonorleucine (Anl) through the expression of a mutant methionyl-tRNA synthetase. Exclusive incorporation of Anl, into native Mtb proteins, provided a click handle to pull out low abundant secretory proteins from the lysates of infected cells. Further, temporal secretome profiling, upon infection with strains of varying degree of virulence, revealed the proficiency of virulent Mtb to secrete chaperones. This ability contributed at least partially to the mycobacterial virulence-specific suppression of ER stress in the host macrophage, representing an important facet of mycobacterial virulence. The Anl labeling approach should facilitate new exciting opportunities for imaging and proteomic investigations of differently virulent Mtb isolates to understand determinants of pathogenicity.

Tuberculosis, the disease caused by *Mycobacterium tuberculosis* (Mtb) continues to claim millions of lives every year. Efforts to develop more effective therapies have seen a resurgence of interest in resolving the interactions between host and pathogen^1^,^2^,^3^. It is now well established that engagement of the host macrophage machinery by the intracellular pathogen plays a critical role in Mtb pathogenesis. Thus, cooption of host cellular function by Mtb facilitates attenuation of anti-microbial responses on the one hand, and also access to essential nutrients on the other^4^,^5^. At least in part these effects are mediated through mycobacterial secretion of a range of protein products that, presumably, interfere with host cellular processes such as signal transduction^6^,^7^,^8^,^9^,^10^,^11^,^12^,^13^. The precise mechanisms by which the Mtb-secreted proteins promote virulence are, however, still unclear and investigations have been constricted by the small number of such proteins that have been characterized so far. While a more comprehensive description of the intracellular Mtb secretome is clearly in order, the technical challenge of distinguishing the few Mtb proteins from the predominant background of the macrophage proteome has posed difficulties.

The recent development of non-canonical amino acid-based bio-conjugation technology has provided a new impetus for the profiling of selective proteomes^14^,^15^,^16^. Notably, the discovery of a new class of methionyl-tRNA synthetase (NLL-metRS) allows genetic incorporation of a methionine surrogate, Azidonorleucine (ANL), in proteins in a cell-selective manner^17^,^18^. Thus, engineering cells to express this mutant form of the enzyme enables residue-specific genetic incorporation of azide functionality (through ANL incorporation) for subsequent tagging of the parent protein with a diverse set of biomolecules. This can be achieved through the copper catalyzed azide-alkyne cycloaddition reaction (Click reaction), a process that is selective, efficient and broad in scope. The Click reaction has been extensively used for the labeling of proteins, glycans, lipids and nucleic acids^19^,^20^,^21^. Thus, ANL functionalization provides a bio-orthogonal handle for selective enrichment of the target proteins through conventional affinity purification protocols. The derivatized proteins can be tagged with biotin, for example, to enable subsequent purification. Alternatively, these proteins can also be directly conjugated to an alkyne-immobilized matrix. Proteolytic digestion on the matrix then releases peptides that can be directly identified by mass spectrometry (MS).

In the present study we extended the NLL-metRS-mediated translational Anl incorporation principle to interrogate the Mtb secretome in infected macrophages. As anticipated, this procedure facilitated selective purification of the relatively low abundant Mtb secretory proteins, from the lysates of infected cells. Subsequent analysis using the label free SWATH-MS platform enabled identification and quantification of the proteins secreted by the intracellular Mtb into the macrophage milieu. Importantly, comparison between the intracellular secretomes of the virulent (H37Rv), an avirulent (H37Ra) and a drug resistant clinical isolate (BND433) helped to identify at least some of the proteins that correlated with mycobacterial virulence. A significant proportion of these proteins belonged to the functional classes implicated in virulence, detoxification, and adaptation pathways.

## Results

### Generation of NLL-MetRS expressing mycobacteria and ANL-labeling of proteins

Recent studies have shown that transgenic expression of NLL-MetRS catalyzes the incorporation of azide functionality in the proteins, facilitating selective labeling in multicellular environments^18^,^22^,^23^. We explored whether engineering Mtb to express NLL-MetRS, an evolutionary conserved enzyme with 75% nucleotide identity, enables differential labeling of Mtb proteins in infected host macrophages. As the first step towards generation of Mycobacterial strains expressing NLL-MetRS gene, we modified the nucleotide sequence in order to adapt it to the Mtb codon usage (codon optimized version hereafter referred as Myco-MetRS; see Supplementary Information for the gene sequence). In addition, HA-tag encoding nucleotides were added to the C-terminus of the encoding sequence to facilitate detection of expression of the recombinant enzyme.

**Figure 1:**
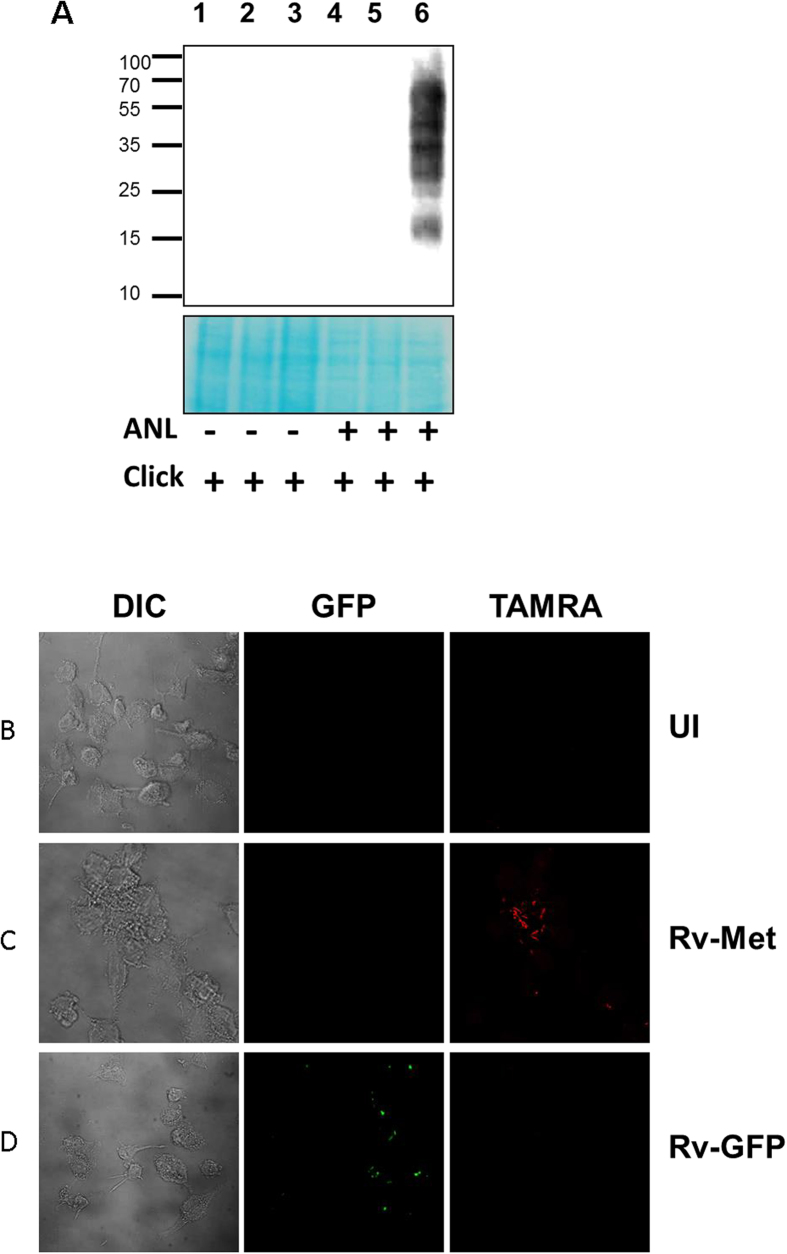
(**A**) Genetic incorporation of ANL in proteins of M. smeg: Cells harboring pMV762 (1 and 4), pMV762-GFP (2 and 5) and pMV expressing Myco-MetRS (3 and6) were grown in the presence (+) or absence (−) of ANL, and all the cell lysates were treated with biotin-alkyne for covalent incorporation using click reaction. Streptavidin–IR800CW dye signal was captured using Odyssey infra-red scanning system. Stained membrane (bottom) served as a loading control. The signal indicates streptavidin interaction with the covalently captured biotin-alkyne to the ANL incorporated into cellular proteins. (**B**–**D**) Fluorescence microscopy to assess strain susceptibility to labeling with TAMRA-alkyne: The differentiated THP-1 cells (**B**); infected with H37Rv expressing either Myco-MetRS (**C**); or GFP (**D**), were maintained in RPMI supplemented with 2 mM ANL for 24 hrs and then checked for incorporation of ANL in proteins. Excess ANL was removed by washing with PBS, cells were fixed and then labeled with TAMRA-Alkyne.

In preliminary experiments we first employed *Mycobacterium smegmatis* (M. smeg) as the target strain to verify that Myco-MetRS could be expressed in a functionally active form. The HA-tagged Myco-MetRS gene or GFP encoding sequence were cloned downstream of the groEL promoter in pMV762 vector (Suppl. Fig. 1A,B), which was then introduced into M. smeg cells by electroporation (Materials and Methods). High levels of GFP expression in the transformed cells verified the efficiency of electroporation (Suppl. Fig. 1C), whereas a Western blot analysis of cell lysates with HA-specific antibody confirmed that the Myco-MetRS protein was indeed being expressed (Supplementary Fig. 1D). To next ascertain the functional activity of the Myco-MetRS enzyme we tested for metabolic incorporation of ANL into the cellular proteins. For this, M. smeg cells transformed either with the empty vector, or vector expressing GFP or Myco-MetRS were pulsed with 1mM ANL for 6hrs, and the corresponding lysates were subjected to a Click reaction with biotin-alkyne. A Western blot analysis of these lysates revealed that biotin incorporation was indeed restricted to the proteins derived from the Myco-MetRS-expressing cells. No biotin labeling was however observed in extracts of M. smeg cells transformed with either the empty- or GFP-carrying vector (Fig. 1A). Thus these results confirm the utility of the NLL-MetRS-based approach in the non-pathogenic mycobacteria, in addition to verifying that the Mtb-specific codon optimization did not compromise enzymatic activity of the protein.

**Figure 2:**
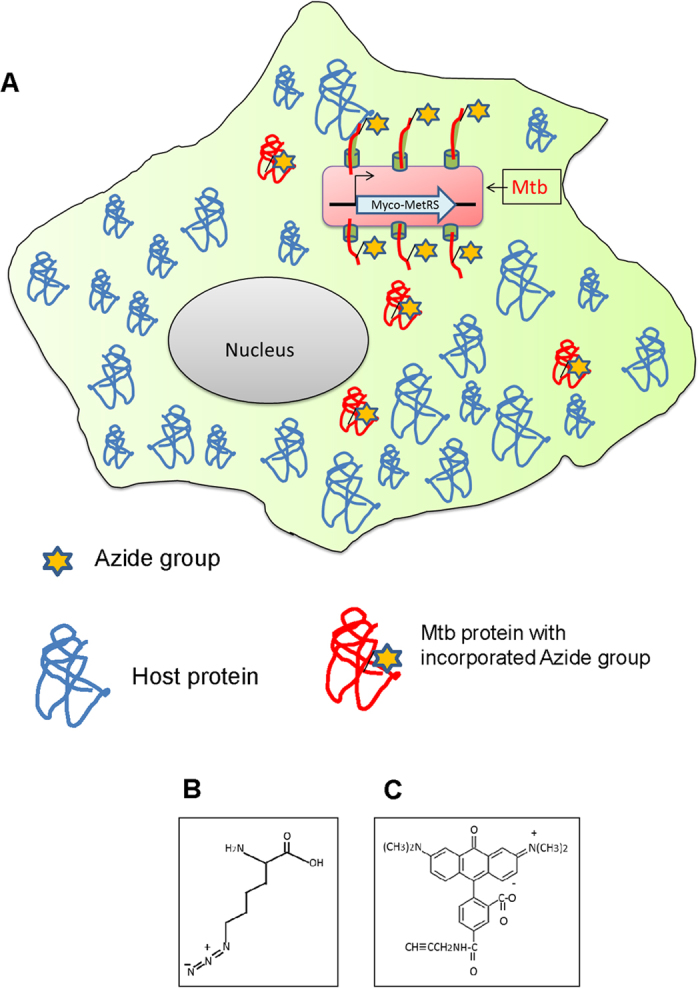
Overview of the method: (**A**) Schematic depicting infected host macrophage with Mtb engineered to express Myco-MetRS for selective proteome labeling through ANL incorporation; structure of ANL (**B**) and TAMRA-Alkyne (**C**).

To further establish the procedure, we next examined the feasibility of introducing Myco-MetRS-encoding plasmid into the virulent mycobacterial strain H37Rv (Rv-Met) for metabolic labeling. Successful electroporation was verified by Western blot experiments confirming the labeling in presence of ANL (Suppl Fig. 2A). Importantly, ANL-pulsing of THP-1 cells infected at an MOI of 10 with Rv-Met, followed by a Click reaction with TAMRA-alkyne, confirmed the expression of labeled proteins in the intracellular environment (Fig. 1C). No such labeling was detected in pulsed - but uninfected (UI) - THP-1 cells, confirming the absence of any non-specific labeling of host proteins by the Click reaction (Fig. 1B). This was also true of cells infected with only a GFP-expressing version of H37Rv (Rv-GFP). While the presence of intracellular bacteria was clearly evident here, protein labeling with TAMRA remained undetectable (Fig. 1D). Thus the results in Fig. 1 established the utility of the Myco-MetRS expression-based approach for selectively detecting Mtb proteins, within infected macrophages. This method will specifically identify the proteins that are being translated during the window of ANL-pulsing because of high specificity of the enzyme towards incorporation of ANL as a surrogate for methionine (Fig. 2).

**Figure 3:**
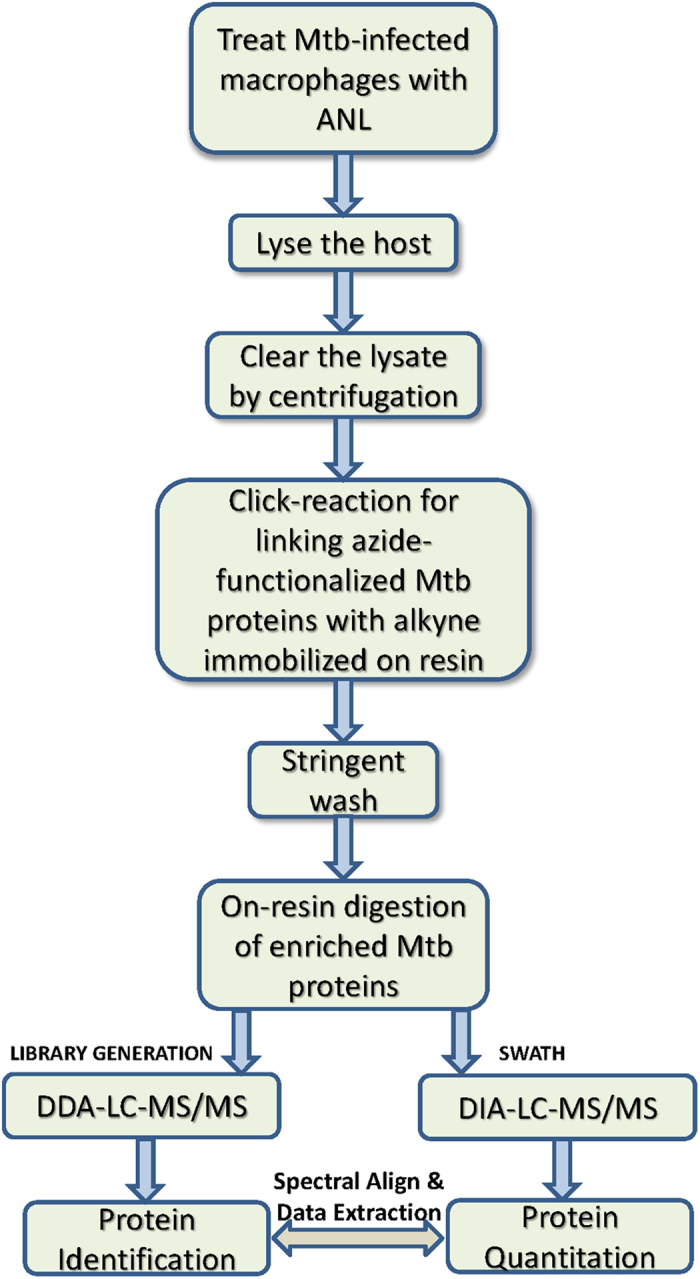
Workflow for the enrichment of Mtb-proteins from infected host macrophages.

We also prepared lysates from parallel batch of THP-1 cells (~100 million) that had been infected for 24 hr with Rv-Met, and the corresponding proteins were then ligated to alkyne-agarose by the Click reaction. The derivatized beads were then subjected to tryptic digestion and the resulting peptides analyzed by liquid chromatography-mass spectrometry (nanoLC-MS) (Fig. 3). Using a stringent cut-off criterion for selection (Methods), samples from three replicates for Rv-Met infected macrophages at 24 hr time point were analyzed and the collective data qualified 44 Mtb-derived proteins (Table 1 and Supplementary Table 1). Importantly, no such proteins were detected in a parallel experiment with lysates from cells infected with the wild type H37Rv (not shown). These results, therefore, establish the specificity of the overall procedure for identifying the Mtb-expressed proteins in THP-1 cells.

### Infectivity and intracellular growth properties of engineered Mtb strains

Since our interest was to interrogate the intracellular Mtb secretome in the context of virulence, we also generated H37Ra and BND433 expressing Myco-MetRS and the labeling was confirmed (Ra-Met, BND-Met; Suppl. Fig. 2B and 2C). While H37Rv and H37Ra are the respective virulent and avirulent counterparts of a laboratory strain, BND433 is a clinical isolate from the CAS lineage^6^,^24^. Thus, given that the mycobacterial panel under study encompasses distinct phenotypic properties ranging from attenuated to different degrees of virulence, a comparative secretome analysis was expected to identify the proteins that may be relevant for pathogenicity. Further, we also felt that a time course profiling of the secreted proteins would prove more informative. Prior to initiating these experiments, however, it was necessary to first verify whether the Myco-MetRS expression in any way affected the capacity of bacteria to either infect THP-1 cells, or grow within them.

**Figure 4:**
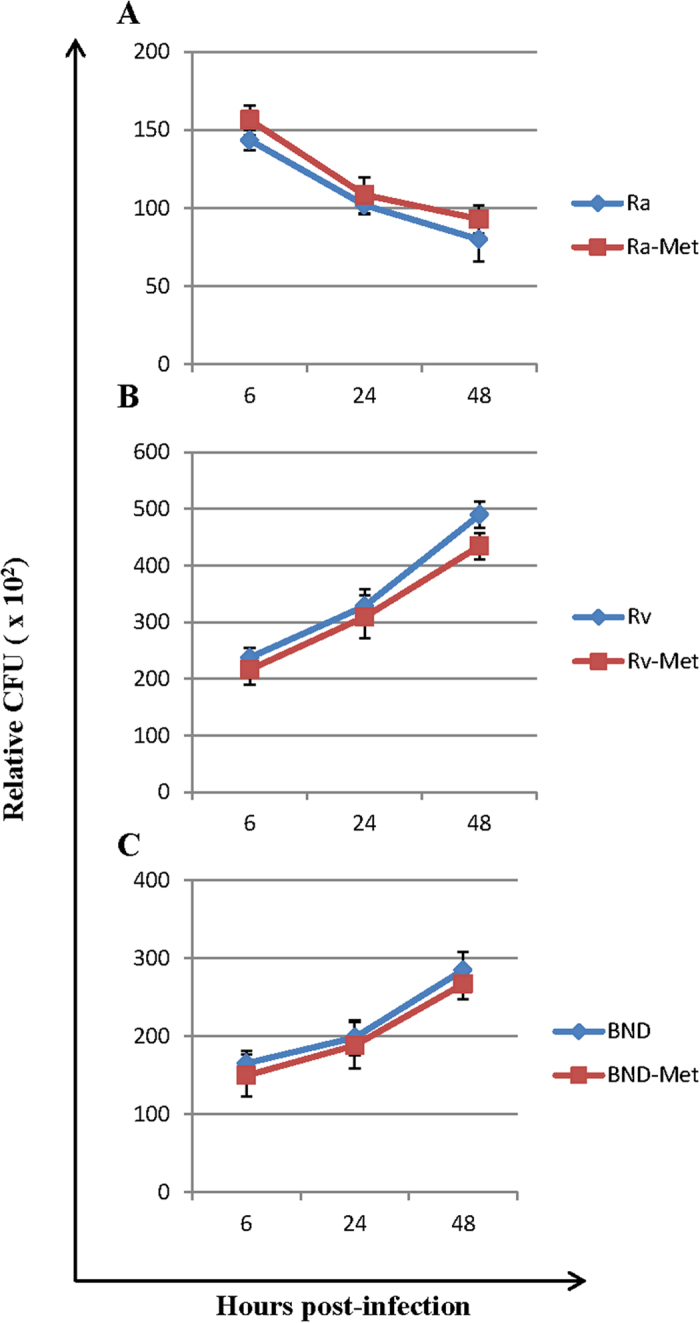
Growth properties of the strains engineered to express Myco-MetRS: (**A**) Ra-Met; (**B**) Rv-Met; (**C**) BND-Met, and their respective wild-type counterparts in infected THP-1 cells. The CFU values, expressed relative to viable host cells, obtained at the indicated times after infection, are shown. Data are the mean (±SD) *n = 3*.

In initial experiments we found that the infection capacity of the Myco-MetRS-expressing strains was somewhat compromised in comparison with that of the wild type counterparts. When both transfected and wild type strains were taken at an MOI of 10, the extent of infection obtained with the former was significantly lower. While the reason for this is not clear at the present time, a two-folder higher MOI for the engineered strains was required for achieving an infection level that was comparable to that obtained with 10 MOI of wild type Mtb. Importantly though, once this correction factor for MOI was applied, both sets displayed a comparable growth profile in the THP-1 cells. This was equally true for all the strains under study (Fig. 4).

We also investigated whether Mtb or the host behave similarly when grown in the presence of ANL. For this, differentiated THP-1 cells were cultured for indicated times in media containing 2 mM ANL and MTT assay was performed to determine the changes in viable host cell number. To assess any changes in Mtb growth, we examined intracellular mycobacterial growth properties in the presence or absence of 2 mM ANL and the bacterial growth was determined by functional colony forming units (CFU) on the 7H11 media at different time points. Although we observed minor differences in growth properties of the pathogen and the host (Supplementary Fig. 3), overall the data demonstrated that cells had little or no effect when compared to grown without ANL. We also examined the possibility that the proteins (Table 1) might have originated from the lysis of bacterial cells. For this, the infected host macrophages were lysed with 1% SDS in PBS (lysis buffer used for lysis before click reaction) and recovered bacteria were again inoculated in 7H9 media. Growth pattern was then determined by OD600 measurement at different time points vis-à-vis its untreated counterparts. Although high viability and similar growth patterns in presence of SDS confirmed the absence of significant cell lysis (Supplementary Fig. 4) and argued against the identified proteins originating from adventitious lysis of the bacterium, we cannot completely exclude the possibility that the minor fraction can originate from the lysis of bacterium. Minor lysis of the bacterium, however, would yield cell-associated Mtb proteins with significant quantity, which was not observed.

### Mass spectrometric analysis of the intracellular secretomes of Mtb strains under study

**Figure 5:**
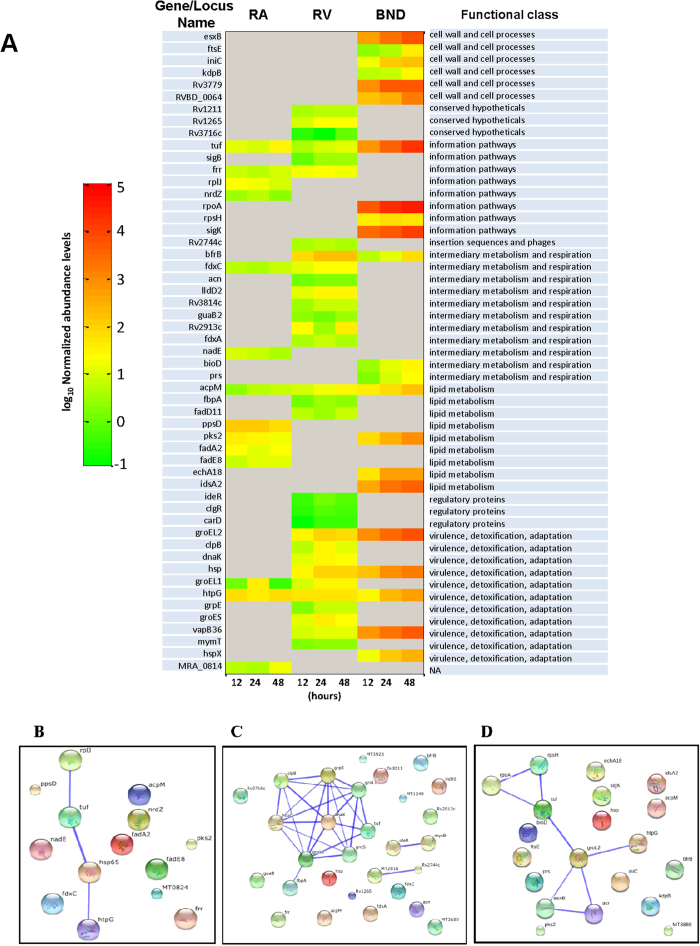
Quantitative temporal profiles of the Mtb-proteins enriched from infected macrophages and network analysis: (**A**) the normalized (to the *E. coli* beta-galactosidase; methods) abundance levels of the secretory proteins obtained by SWATH are plotted as a heat map. The proteins secreted at indicated time points by H37Ra, H37Rv and BND433 strains are shown. The proteins are grouped manually according to TubercuList functional category. (**B**–**D**) The protein-protein interaction network obtained from STRING database for the interaction among the secretory proteins of H37Ra (**B**), H37Rv (**C**) and BND433 (**D**). The stronger associations among the molecules are shown by thicker edges. Refer Supplementary Table-2 for a list of information obtained from STRING database.

THP-1 cells were infected, in the presence of ANL with Rv-Met, Ra-Met or BND-Met for 12, 24, or 48 hrs and Mtb proteins were enriched following a workflow as outlined in Fig. 3. At each of these time points, cell lysates were prepared from all the sets and the ANL-incorporating proteins were coupled to alkyne-agarose beads through the Click-reaction. Bound proteins were digested with LysC and trypsin, and the resulting peptides were identified by nanoFlex cHiPLC-MS/MS. For the quantitative proteomic analysis we employed label-free SWATH-MS platform for absolute quantitation that combines high specificity data independent acquisition (DIA) with a novel targeted data extraction strategy to mine the resulting fragment ion data sets (refer Methods). Mycobacterial protein abundance was quantified from 20 × 10^6^ differentiated THP-1 macrophages at each of the time points. By using high-confidence filter criteria (Methods; Supplementary Fig. 5), samples from three biological replicates for all Mtb strains at each time point were analyzed and the collective data quantified 15, 31 and 22 proteins to be secreted by H37Ra, H37Rv and BND433 respectively. Of these, 6 were common to H37Ra and H37 Rv while 7 proteins were common between H37Rv and BND (Fig. 5A; Supplementary Table 2). Although almost all the proteins identified in this study comprised the list of Mtb culture filtrate proteins reported in an earlier study^25^, some of the proteins like VapB36, Rv3814c in case of H37Rv; MRA_0814 in case of H37Ra; and Rv3779, VapB36, RVBD_0064 in case of BND433 were not identified as a part of Mtb secretome in an earlier report^25^. However, to further study these proteins require refined targeted genetic approaches to understand the possible role of these virulent factors in establishing the infection.

Figure 5A gives the time course profile of expression of these proteins, where the latter have been grouped in accordance with their functional classification in the TubercuList^26^. From the profile it could be seen that majority of proteins secreted by H37Rv and BND showed a relative increase in abundance over the infection period. The only exceptions to this were Frr, FdxA, FbpA, and ClpB, in case of H37Rv whose intracellular levels decreased by 48 hrs post-infection. Conversely, levels of the Mtb-secretory proteins in H37Ra infected cells showed a general decline over time in all cases except Tuf, Frr, AcpM, FadE8 and MRA-0814. It is possible that these distinct overall profiles reflect the corresponding growth profile curves in Fig. 4. Frr, however, presents an interesting case in this context. Although being present in both H37Ra and H37Rv its concentration decreased with time in H37Rv-infected cells, whereas the opposite was true in the case of H37Ra infection. Admittedly though, the temporal abundance of Mtb secretory proteins (when hypervirulent, virulent and avirulent strains are compared) do not exhibit a simple linear relationship but are complex, and will likely require in-depth biochemical studies to understand its biological significance.

Classification of the secretory proteins according to their functional category revealed that 10 out of 31 proteins secreted by H37Rv and 5 out of 22 proteins secreted by BND433 were indeed involved in virulence, detoxification, and adaptation (Fig. 5A). They all belonged to the family of chaperones/heat shock proteins. In contrast only 2 of the 15 proteins identified from H37Ra-infected cells, HtpG and GroEL1, were from this functional class. Interestingly though, both of these proteins were commonly present in cells infected with either of the laboratory strains. The other major functional classes of the H37Rv secretory proteins included information pathways, metabolism, and regulatory functions (Fig. 5A). Moreover, BND433 infection also accompanied by the release of a class of proteins involved in cell wall and cell process. Significantly, extraction of a network of interactions by these secretory proteins using the STRING database^27^ revealed that the chaperone proteins formed a tightly connected cluster with high confidence (score > 0.7) (Fig. 5C). This is suggestive of an inter-dependent mode-of-action between these proteins in case of H37Rv, which may have relevance during the process of infection. Indeed, chaperones have classically been identified to act in concert in infection systems, so as to reduce cellular stress^28^,^29^. Evidence also suggests that chaperones facilitate efficient mycobacterial association with macrophages and polarizing in M2-like phenotype, indicative of an extracellular role^30^,^31^. Kruh-Garcia *et al.* recently documented enrichment of HspX, BfrB, KatG, FbpA, GroEL2, DnaK, AcpM, and GroES in the exosomes of infected patients, suggesting the extracellular function of these virulent proteins during the establishment and maintenance of intracellular infection^32^. Interestingly though, majority of these proteins enriched in the exosomes were present in cells infected with H37Rv and BND433 strains, suggestive of their relevance during the process of infection. Furthermore, the functional class of proteins identified in the case of H37Ra majorly belonged to lipid metabolism, information pathways, and virulence (Fig. 5A). However, the network of interactions obtained in this case was very sparsely connected (Fig. 5B), indicating a lack of a concerted functioning of these proteins. While in case of BND network depicted interaction between chaperones and cell wall proteins, proteins involved cell processes and information pathways (Fig. 5D).

Thus, at one level, our results highlight that infection of macrophages with a virulent Mtb strain is accompanied by release of a significantly larger number of chaperones, relative to the situation with H37Ra infection. Further, although HtpG and GroEL1 were common to both H37Ra and H37Rv, the temporal abundance profile of GroEL1 differed significantly. In the case of H37Ra infection, this protein was found to appear only transiently at the 24 hr time point. As opposed to this, while GroEL1 was also induced only after 24 hr of infection with H37Rv the levels, however, persisted up to at least 48 hr (Fig. 5A). This distinction in the GroEL1 (hsp65) abundance profile between H37Ra and H37Rv infected cells prompted us to probe the potential role that it might play in facilitating the intracellular survival of Mtb. Importantly, given that the H37Rv-secreted chaperones form a tightly connected cluster, we anticipated that such a study would also provide more general insights, on the function of these proteins in the context of mycobacterial virulence.

**Figure 6:**
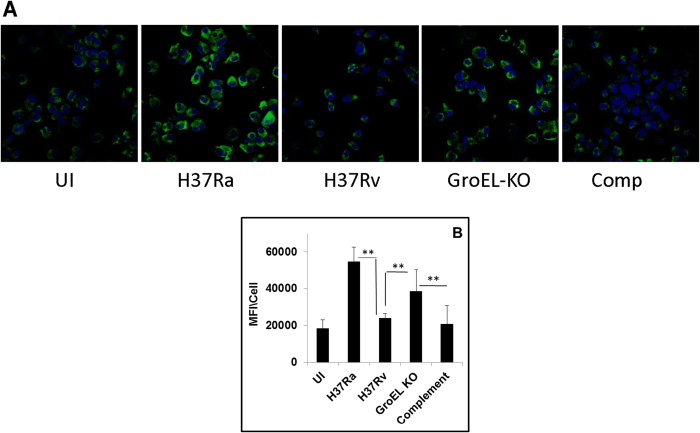
The ER stress response of the uninfected (UI) macrophages and macrophages infected with the indicated panel of strains: (**A**) representative images of ER stress documented by probing an ER stress marker (Grp78) and visualized with secondary Alexa488 conjugated antibody (green). Nuclei were counterstained with Hoechst; (B) Graph represents the quantification of Grp78 from three independent experiments, with at least 100 cells scored from each field. (**pValue ≤ 0.001, calculated with paired t-tests).

Given the known role of mycobacterial GroEL in both assisting protein folding, as well as in suppressing aggregation of unfolded proteins, we speculated that GroEL1 may function in the host macrophage by suppressing the cellular ER stress response and, thereby, prevent the activation of apoptosis. Several earlier studies have demonstrated that suppression of host cell apoptosis represents an important facet of mycobacterial virulence^33^,^34^,^35^,^36^,^37^. To test this we separately infected THP-1 cells with either H37Ra, H37Rv (the respective virulent and avirulent counterparts), a GroEl1-knockout variant of H37Rv (GroEL-KO), or GroEL1-KO that had been complemented with the GroEL1 gene (Comp)^38^. Infection was for 24 hrs, after which cells were stained for the ER stress marker Grp78 and then visualized by confocal microscopy. Whereas H37Ra infection produced significant ER stress in the host cells this, however, was not the case in H37Rv-infected cells (Fig. 6). That is, H37Rv appeared to be capable of suppressing the ER stress response of the host cell to infection. Interestingly though, this ability to suppress ER stress was partially diminished in the GroEL1-KO bacteria, although complementation with the GroEL1 gene (Comp) then again fully restored this capacity (Fig. 6). Collectively, therefore, these results confirm that GroEL1 contributes at least partially to the mycobacterial virulence-specific suppression of ER stress in the host cells. It is highly likely here that the partial effect seen with GroEL1-KO may be due to at least some degree of functional redundancy with the other nodes of the chaperone network (Fig. 5B,C). Similarly, although GroEL1 is transiently produced in H37Ra-infected cells, its activity could likely be muted because of the absence of the other chaperones that may contribute to its function. Both of these aspects, however, remain to be experimentally verified.

## Discussion

Although efforts have been made in studying the proteome of the pathogen in broth culture and cell-free supernatant^25^,^39^,^40^, little is known about the nature and composition of the proteins that are secreted by mycobacteria once they are inside the host macrophage. This latter aspect is especially relevant considering that successful infection involves time-dependent manipulation of a series of host defense mechanisms, which threaten bacterial survival. Virulent Mtb strains, however, have evolved strategies to counter host antimicrobial responses, thereby successfully establishing a secure niche within the host intracellular milieu. The dynamics of host-pathogen crosstalk, thus takes a central stage in dictating the outcome of the infection process.

The highlight of our present report is the description and validation of a new approach that enables study of the intracellular Mtb-secretome. Importantly, we also demonstrated the utility of this approach in mapping the temporal profiles of secretion of the various protein components, in addition to comparing such profiles between differently virulent Mtb strains. As shown here, a comparison between H37Ra, H37Rv and BND 433 identified proteins selectively secreted by the virulent strains.

Although the functional relevance of most of these proteins remains to be investigated, our preliminary results suggest that the secreted chaperones contribute to supporting bacterial survival, at least in part, by modulating the ER stress response of the host cell. The activation of an apoptotic response by the host macrophage represents an innate defense mechanism that restricts microbial spread and enhances the induction of adaptive immunity^12^,^37^. Virulent Mtb strains, however, are known to inhibit apoptosis in the early stages of infection. This serves as a key strategy for enabling establishment of infection in the host^12^,^33^. Thus, the chaperone-mediated buffering of host cellular ER stress could well constitute one of the pathways by which the pathogen achieves this objective. The link between ER stress and apoptosis has been well documented in the literature^41^,^42^,^43^.

However, the method set the stage for further validation of protein identities such as employing more knockout strains or pathogen expressing the tagged proteins to verify the secretion and identification of the location inside the host macropahges. While some (or perhaps many) proteins may have been released as a consequence of unintended bacterial lysis, the method might improve with further investigations towards better host cell lysis techniques. The absence of many of the reported and established secretory proteins in the SWATH data could be attributed to 1. The amount of starting material, 2. A prerequisite chromatography separation platform used for SWATH 3. More number of steps involved in sample handling or simply, 4. Abundance of these proteins in the sample.

Nevertheless, the method capitalizes further on alkyne functional group immobilized on resin (alkyne-agarose) to avoid large number of steps involved and consequent loss of proteins due to former (Fig. 3), thus facilitating the purification, on-bead digestion and subsequent identification by MS. Moreover, with SWATH analysis we show that this method can be easily combined with label-free platforms for absolute protein quantitation with further room for iTRAQ and SILAC based approaches where multiple strains can be profiled at the same time in a single reaction tube. We show that method is ideally suitable for studying not only qualitative protein profiles of the Mtb strains but to interrogate also the transient changes in the intra-macrophage Mtb-protein abundance as a function of time, with further avenues for improvement to study infection dynamics in animals.

In conclusion, we recognize that the list of proteins identified here is by no means exhaustive. Nonetheless, our findings set the stage for a more detailed description of the complement of proteins secreted by Mtb in the host macrophage. Such studies then, coupled with a functional analysis of the roles of these proteins in the intracellular milieu, should yield novel insights into mechanisms governing mycobacterial pathogenesis.

## Materials and Methods

### Mycobacterial expression plasmid

*E. coli* expression plasmid pJTN5 containing NLL-MetRS was a kind gift from David Tirrel^18^. The sequence obtained from pJTN5 for NLL-MetRS encoding gene was used as a template and then codon usage was adapted to the codon bias of Mtb genes. Mycobacterial expression optimized variant of NLL-MetRs sequence was synthesized by GeneArt (Life Technologies-USA). The optimized MetRS ORF (Myco-MetRS; Supplementary information) was further PCR amplified from the respected supplied clones with the primers incorporated to have NcoI/HinDIII sites and the C-terminal HA tag [NcoI_Met-F: ATTCCATGGCCACCCAGGTGG CCAAG; HA_Met-R1: TAAGCTTTCAGCCGGCGTAGTCCGGCACGTCGTACGGGTACATGCCCT--TCACCTGGTGACCCGGTTTGGCAC; HinDIII-HA_Met-R2: TATAAGCTTTCAGCCGGC--GTAGTCCGGCACGTCGTACGGGTACATGCCCTTCACCTGGTGACCCGGT].

The amplified product was cloned in pTZ57R (Fermentas- Thermo Scientific, USA) by T/A cloning and recombinants were screened using NcoI and HinDIII sites. Subsequently, the ORF was released from pTZ and cloned downstream of hsp60 promoter into Ecoli-Mycobacteria shuttle vector, pMV762^44^ at identical sites of the vector (Suppl. Fig. 1A). GFP encoding nucleotides were amplified from pEGFPN2 (Clontech, USA) with primers incorporated to have identical RE sites [NcoI_GFP-F: ATT CCA TGG TGA GCA AGG GCG AGG AGC TG; HinDIII_GFP-R: TAT AAG CTT TTA CTT GTA CAG CTC GTC CAT G], cloned in pTZ57R, and sub-cloned at identical sites of pMV762 (Suppl. Fig. 1B).

### Bacterial strains and culture conditions

H37Ra, H37Rv, were obtained from A. Tyagi (Delhi University), and *M smegmatis* from A. Singh ICGEB. Bacteria were grown in Middlebrooke 7H9 broth (Difco) supplemented with 10% ADC (Becton Dickinson), 0.4% glycerol and 0.05% Tween-80 until the mid-log phase. The bacteria were harvested, washed with RPMI and re-suspended in the same media. Single cell suspension for infection assays was obtained by dispersion through a 30-guage needle. Bacteria were quantified by measuring the absorbance at a wavelength 600 nm. Detailed protocols and culture conditions are described elsewhere^6^,^24^.

### Electroporation

Log phase cultures were used for electroporation. Briefly, bacterial cultures were grown until OD 600 reaches to 0.6, in 7H9 medium supplemented with 10% ADC supplement. Cells were harvested by centrifugation for 10 mins at 4000 x g. Supernatant was decanted and cell pellet was re-suspended in 10 ml of 10% glycerol and spun at 4000 x g/10 mins. The pellet was again washed with 5 ml of glycerol and cells were spun at 3000 x g/10 mins. Finally, the pellet was re-suspended in 1 ml of glycerol and either used immediately or stored at −80 °C for further use. All the centrifugation steps were carried out at 4 °C and cells were kept on ice. Electroporation cuvettes (0.2 mm BioRad) were pre-chilled on ice and 300 μl cell suspension was then added to the cuvettes and mixed with DNA (5 μg). For 0.2 mm cuvettes were given pulse at 2.5 kv/resistance 1000 Ω. Cells were immediately replenished with 3 ml fresh growth medium and revived for 3–4 hrs (for M. smeg) or 24 hrs for Mtb. After revival the transformed cultures were spun and pellet was re-suspended in 150 μl of growth medium and plated on a (50 μg/ml) hygromycin containing 7H11 agar plate.

### Cell culture and Infection

Human THP-1 monocytic cell line was maintained in RPMI supplemented with 10% FBS (Hyclone) and 50 μg/ml PENSTREP (Gibco) in a humidified incubator at 37 °C with 5% CO_2_. PMA (30 ng/ml) differentiated THP-1 cells were infected with Mtb at a multiplicity of infection of 10:1 or 20:1 over a 4 hours period. This was followed by Amikacin treatment for 2 hrs to remove any extracellular bacteria as described earlier^24^.

### Immunoblotting

Total proteins were precipated by 10% TCA followed by washing (2x) the precipitates with cold acetone. Pellets were re-suspended in Laemmli buffer and proteins were separated on 10% SDS-PAGE. The resolved proteins were then transferred onto nitrocellulose membrane and blocked with 1X odyssey blocking buffer in PBS. Subsequently, biotinylated proteins were detected using Streptavidin Licor 800CW using Odyssey infra-red imager. For loading reference, membranes were stained using reversible membrane staining kit (Pierce; Thermo Scientific).

### Microscopy

Briefly, 15,000 differentiated THP1 cells were infected with ANL-pulsed H37Rv at 10MOI in a glass bottom 96 well plate (Nunc, Denmark), as described above. The infected cells were maintained at 2 mM ANL and washed twice with PBS (pH7.8) after 24 h incubation and fixed with 4% paraformaldehyde for 30 mins at 37 °C. Fixative was removed by washing (5 times) with PBS and cells were permiabilized with 0.1% triton-X-100 at RT for 3 mins and washed thrice with PBS. After washing cells were resuspended in PBS and dye labeling was initiated by adding 200 μM triazole ligand, 400 μM of tris(2-carboxyethyl)phosphine hydrochloride, 200 μM CuSO4, and TAMRA-Alkyne (Molecular Probes-Invitrogen, USA) diluted to 1:1000 as obtained from the manufacturer. Dye labeling was allowed to proceed for 16 h at RT in dark with gentle agitation. After incubation, the cells were rinsed three times with PBS containing 0.5 mM EDTA and 1% Tween-20. Cells were covered with prolong Gold Antifade (Invitrogen) and imaged using the confocal microscope. The GFP expressing H37Rv was used as a control and the samples were processed identically to assess the non-specificity.

For immunofluorescence, the infected cells were fixed identically and Grp78 expression was detected using primary monoclonal antibody to Grp78 (Abcam, USA) followed by secondary antibody labeled with Alexa488. Nuclei were counterstained with Hoechst. Cells were observed with a Nikon Eclipse Ti-E laser scanning confocal microscope equipped with 603/1.4 NA PlanApochromat DIC objective lens.

### Protein pulse labeling and conjugation

Cells were grown in 7H9 medium supplemented with ADC at log phase (OD00 = 0.5) and pulsed with 1 mM ANL at indicated time points prior to conjugation to alkyne. A culture of Mtb strains where no ANL was added was used as a control. Cells were harvested by centrifugation at 4000 x g at RT for 5 mins, washed with PBS (pH7.8) twice and lysed in PBS with 10%SDS at 90 °C for 15 m. The clarified lysates were diluted with PBS to the final SDS concentration of 1% and ANL-labeled proteins were then conjugated with biotin by addition of 200 μM triazole ligand, 25 μM biotin alkyne, and 400 μM CuBr; overnight at room temperature.

### Enrichment of Mycobacterially secreted proteins from infected host macrophages, on-bead digestion and sample preparation for mass spectrometry

ANL susceptible strains were pulsed 12 h before infection and used to infect differentiated macrophages in RPMI medium as described above. The cells were harvested at indicated time points for Azide-labeled secretome enrichment by covalent capture using Click-iT Protein Enrichment Kit (Cat#10416; Molecular Probes-Invitrogen, USA). Briefly, Mtb-infected cells were washed with PBS and lysed using 500 μl of 1% SDS supplemented with EDTA-free protease inhibitors (Roche; Complete EDTA-free protease inhibitor cocktail). Bacteria were removed from the lysate by centrifugation at 11,000 × *g* for 10 mins at RT and the lysate was further collected clarified in a fresh tube by adding 100 units of Benzonase (Sigma, USA). Click-Reaction was performed by diluting the cleared lysate to 1 mL with lysis buffer following the vendors’ protocol. After incubation for 16 h at RT the resin was washed with 1.8 ml of 18 mΩ water. Proteins were reduced in 1mL SDS buffer containing 100 mM DTT (Sigma, USA) and incubated at 70 °C for 15 mins. The supernatant was aspirated to waste, and enriched proteins were alkylated in SDS wash buffer containing 7.4 mg/mL of iodoacetamide (Sigma, USA) in dark for 30 mins. The resin was transferred to a spin column (Bio-Rad) and washed with 20 mL of SDS wash buffer, 20 mL of 8 M urea in 100 mM Tris (pH 8), and 20 mL of 20% acetonitrile (Fluka). For release, resin bound proteins were digested with 0.5 μg LysC (Sigma) and 0.5 μg Trypsin and incubated overnight at 37 °C. The peptide solution was collected by spinning down the resin and transferred to a fresh tube and the resin was washed with 500 μl of MS grade water and supernatants were combined. The digest was further acidified with 2 μl formic acid and desalted on a C-18 cartridge. The desalted digest was then used for MS acquisition.

### LC-MS/MS analysis

All samples were analyzed by reverse-phase high-pressure liquid chromatography electrospray ionization tandem mass spectrometry (RP-HPLC-ESI-MS/MS) using an NanoLC-Ultra 1D plus (Eksigent; Dublin, CA) and nanoFlex cHiPLC system (Eksigent) which is directly connected to an ABSCIEX 5600 Triple-TOF (AB SCIEX; Concord, Canada) mass spectrometer, referred as Triple TOF system.

Reverse Phase -HPLC was performed via a trap and elute configuration using Nano cHiPLC columns (Eksigent); the trap column (200 μm × 0.5 mm) and the analytical column (75 μm × 15 cm) were both manufacturer (Eksigent)-packed with 3 μm ChromXP C-18 (120 Å). Reverse-phase LC solvents included: mobile phase A: 2% acetonitrile/98% of 0.1% formic acid (v/v) in water, and mobile phase B: 98% acetonitrile/2% of 0.1% formic acid (v/v) in water. The auto-sampler was operated in full injection mode overfilling a 1 μl loop with 3 μl analyte for optimal sample delivery reproducibility. All samples were eluted from the analytical column at a flow rate of 300 nL/min using a linear gradient of 5% solvent B to 60% solvent B over duration of 60 min, linear 60–90% for 2 min. The column was regenerated by washing with 90% solvent B for 6 min and re-equilibrated with 5% solvent B for 20 min. Autocalibration of spectra occurred after acquisition of every 2 samples using dynamic LC–MS and MS/MS acquisitions of 25 fmol β-galactosidase.

Mass spectra and tandem mass spectra were recorded in positive-ion and “high-sensitivity” mode with a resolution of ~35,000 full-width half-maximum. Peptides were injected into the mass spectrometer using 10 μm SilicaTip electrospray PicoTip emitter (New Objective Cat. No. FS360-20-10-N-5-C7-CT), and the ion source was operated with the following parameters: ISVF = 2100; GS1 = 20; CUR = 25.

Samples used to generate the SWATH-MS spectral library were subjected to conventional, data-dependent acquisition (DDA). The data acquisition mode in DDA experiments was set to obtain a high resolution TOF-MS scan over a mass range 350–1250 m/z, followed by MS/MS scans of 20 ion candidates per cycle with activated rolling collision energy, operating the instrument in high sensitivity mode. The selection criteria for the parent ions included the intensity, where ions had to be greater than 150 cps, with a charge state between +2 to +5, mass tolerance of 50 mDa. Once an ion had been fragmented by MS/MS, its mass and isotopes were excluded from further MS/MS fragmentation for 12s. Collision-induced dissociation was triggered by rolling collision energy. The ion accumulation time was set to 500 ms (MS) and to 200 ms (MS/MS).

For SWATH MS-based experimental samples, the instrument was tuned to specifically allow a quadrupole resolution of 30 Da/mass selections. An isolation width of 30 Da was set in a looped mode over the full mass range (350–1250 m/z) scan and 31 overlapping windows were constructed. For these experiments, the mass spectrometer was operated such that a 250-ms survey scan (TOF-MS) was performed and subsequent MS/MS experiments were performed on all precursors. These MS/MS experiments were performed in a cyclic manner using an accumulation time of 96 ms per 30-Da swath (31 swaths total) for a total cycle time of 3 s. Ions were fragmented for each MS/MS experiment in the collision cell using rolling collision energy.

### Generating the Reference Spectral Library

All DDA mass spectrometry files was searched in Protein Pilot software v. 4.5 (AB SCIEX, Revision- 1656) with the Paragon algorithm. For Paragon searches, the following settings were used: Sample type: Identification; Cysteine Alkylation: methyl methanethiosulfonate (MMTS); Digestion: Trypsin; Instrument: TripleTOF5600; Special Factors: BONCAT Workflow; Species: Mycobacterium tuberculosis complex; Search effort: Thorough ID; Results Quality: 0.05. Only peptides with a confidence score of >0.05 were considered for further analysis. The search was conducted using a through identification effort of a UniProt Swiss-Prot database (April 2014 release) containing species specification. False discovery rate analysis was also performed. The output of this search is a group file and was used as the reference spectral library. The group file contains the following information that is required for targeted data extraction: protein name and UniProt accession, cleaved peptide sequence, modified peptide sequence, relative intensity, precursor charge, unused Protscore, confidence, and decoy result. The parameters used for identification of proteins includes: 1) Threshold of 5% accepted Global False discovery rate (G-FDR) proteins; 2) At least one unique peptide with 95% confidence. The false positive rates of the aforementioned filter criteria were all below 5%, estimated by using an individual reversed (decoy) sequence database^33^.

### Spectral Alignment and Targeted Data Extraction

Spectral alignment and targeted data extraction of Data Independent Acquisition (DIA) samples were performed using PeakView v.1.2 (AB Sciex) using the reference spectral library. For the extraction of data acquired in SWATH mode, an ion library for mycobacterium tuberculosis was generated from data acquired in data dependent mode (spectral data were acquired in DDA mode and analysed using the Paragon search strategy as described above). Datasets from SWATH MS/MS acquisitions were processed using the full scan MS/MS filtering module for data-independent acquisition within PeakView v.1.2 (AB Sciex) and Marker view v.1.2.1 (AB Sciex) software.

All DIA files were loaded and exported in txt format using an extraction window of 10 min and the following parameters: 2 peptides, 3 transitions, peptide confidence of >95%, exclude shared peptides, and XIC width set at 50 ppm. This export results in the generation of three distinct files containing the quantitative output for (1) the area under the intensity curve for individual ions, (2) the summed intensity of individual ions for a given peptide, and (3) the summed intensity of peptides for a given protein. This protein data was used for all data analysis and is provided in Supplementary Table 2. The reversed sequences were removed from the data set prior to further analysis.

## Additional Information

**How to cite this article**: Chande, A. G. *et al.* Selective enrichment of mycobacterial proteins from infected host macrophages. *Sci. Rep.* **5**, 13430; doi: 10.1038/srep13430 (2015).

## Supplementary Material

Supplementary Information

Supplementary Information

Supplementary Information

## Figures and Tables

**Table 1:** H37Rv-secretory proteins enriched from the infected host macrophages.

S.No.	Gene name	Protein name	Found in Kelkar *et al*., 2011.
1	dnaK	Chaperone protein	Yes
2	RVBD_0384c	Endopeptidase ATP binding protein chain B ClpB	Yes
3	hsp	Heat shock protein Hsp	Yes
4	groL	60 kDa chaperonin	Yes
5	RVBD_3841	Bacterioferritin BfrB	Yes
6	esat6	6 kDa early secretory antigenic target	Yes
7	sufC	ABC transporter ATP-binding protein	Yes
8	RVBD_2744c	Alanine rich protein	Yes
9	cfp-10	10 kDa culture filtrate protein	Yes
10	RVBD_0968	Uncharacterized protein	Yes
11	groS	10 kDa chaperonin	Yes
12	clpP	ATP-dependent Clp protease proteolytic subunit	Yes
13	RVBD_2031c	Heat shock protein HspX	Yes
14	RVBD_3583c	CarD family transcriptional regulator	Yes
15	RVBD_0467	Isocitrate lyase Icl	Yes
16	grpE	Protein GrpE	No
17	RVBD_1464	Cysteine desulfurase	Yes
18	Rv2159c	Alkylhydroperoxidase AhpD family core domain-containing protein	Yes
19	RVBD_3131	Uncharacterized protein	Yes
20	RVBD_2710	RNA polymerase sigma factor	Yes
21	sigE	Isoform 3 of ECF RNA polymerase sigma factor	Yes
22	RVBD_2476c	NAD-dependent glutamate dehydrogenase	Yes
23	prpC	Citrate synthase	No
24	groEL	60 kDa chaperonin	Yes
25	katG	Catalase-peroxidase	Yes
26	RVBD_0046c	Myo-inositol-1-phosphate synthase Ino1	Yes
27	prpD	2-methylcitrate dehydratase	No
28	tuf	Elongation factor Tu	Yes
29	vapC	Ribonuclease	Yes
30	Rv0787	Uncharacterized protein	Yes
31	Rv1211	Conserved protein	Yes
32	RVBD_3914	Thioredoxin	Yes
33	rpmE	50S ribosomal protein L31	Yes
34	acpP	Acyl carrier protein	Yes
35	Rv0810c	Uncharacterized protein	No
36	secE2	Calcium dodecin	Yes
37	PE_PGRS50	PE-PGRS family protein PE_PGRS50	Yes
38	Rv0922	Possible transposase	No
39	RVBD_2503c	Succinyl-CoA:3-ketoacid-CoA transferase beta subunit ScoB	Yes
40	rpoC	DNA-directed RNA polymerase subunit beta	Yes
41	Rv2864c	Penicillin-binding lipoprotein	Yes
42	rpoA	DNA-directed RNA polymerase subunit alpha	Yes
43	Rv0449c	Uncharacterized protein	No
44	aceAb	Isocitrate lyase	Yes
