# Supplementary material for: Selective enrichment of mycobacterial proteins from infected host macrophages
Source: Sci Rep. 2015 Aug 25;5:13430. doi: 10.1038/srep13430 (PMC4548221; doi:10.1038/srep13430)
Supplement: Supplementary Information [file srep13430-s1.pdf]

Supplementary information

Selective enrichment of mycobacterial proteins from infected host macrophages

Ajit G. Chande[1,2#], Zaved Siddiqui[1], Mukul Kumar Midha[3], Varsha Sirohi[1], Srikanth Ravichandran[1] and Kanury V. S. Rao[1,3]

[1]Immunology Group, International Centre for Genetic Engineering and Biotechnology, New Delhi-India
[3]Drug Discovery Research Centre, Translational Health Science and Technology Institute, Gurgaon, Haryana-India

[#] **Correspondence**: ajit.chande@gmail.com
[2] **Current address:** Centre for Integrative Biology-University of Trento, Italy.

Supplementary Fig. 1

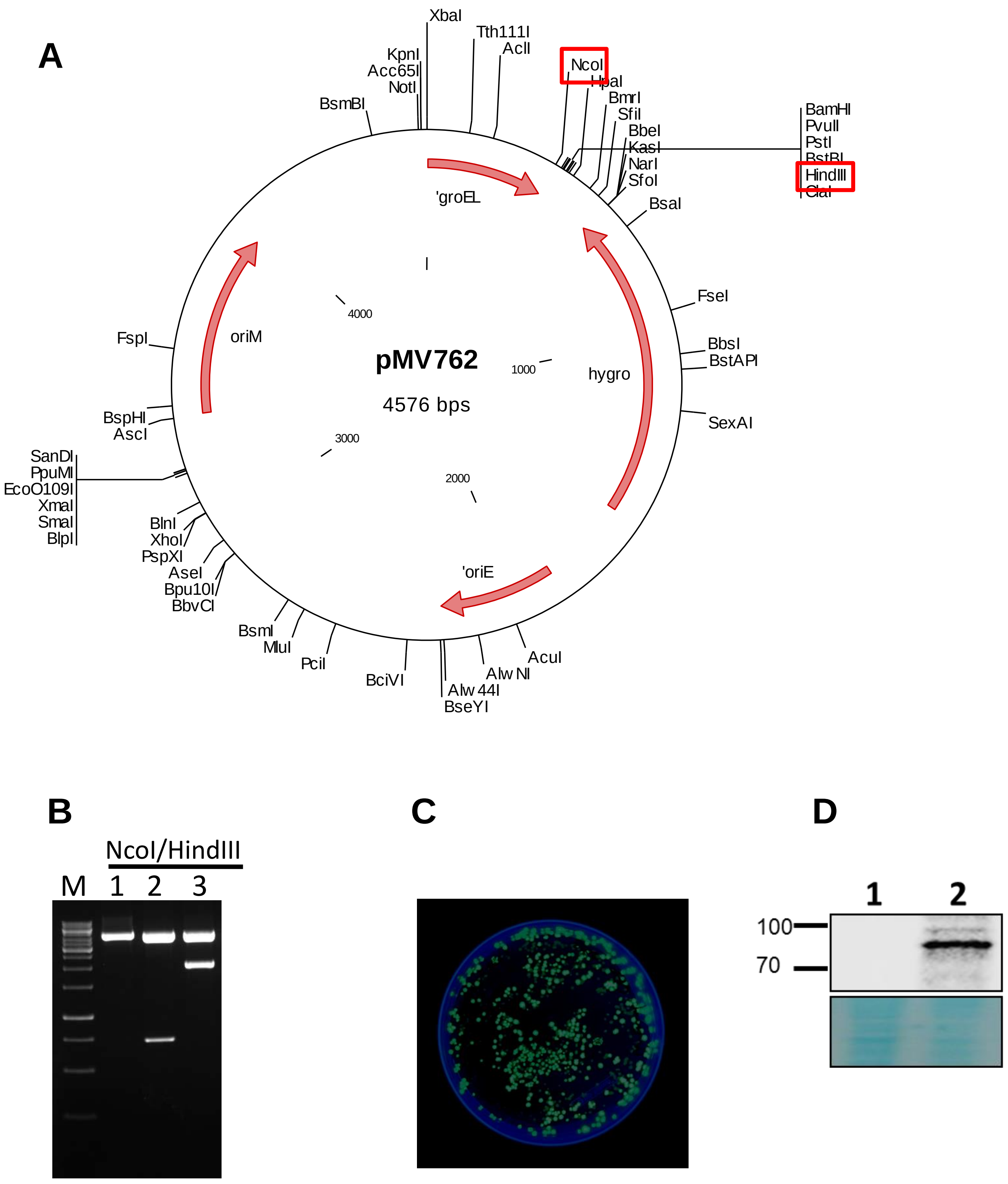

1: pMV762

2: pMV-GFP

3: pMV-Met-HA

M: 1kb DNA marker

Suppl. Fig-1. Generation of mycobacterial constructs for expression of the target protein and metabolic labeling: (A) pMV762 vector map showing the NcoI/HindIII sites downstream to the GroEL promoter; (B) The GFP and HA-tagged Myco-Met encoding nucleotides were cloned in pMV762 at NcoI/HindIII sites. The gel picture shows the fragment release upon digestion with the said enzymes; (C) GroEL promoter driven GFP expression in M. smeg colonies: pMV762-GFP vector transformed colonies were selected on hygromycin and checked for the expression of GFP under a UV trans-illuminator; (D) HA tagged Myco-MetRS expression from the lysates of empty vector transformed (1) and pMV-Myco-MetRS-transformed (2) M. smeg strain was assessed by immunoblotting using mouse monoclonal anti-HA antibody (1:4000 dilution). The signal was detected using IR-labeled anti-mouse secondary antibody. Stained membrane served as a loading control.

Supplementary Fig. 2

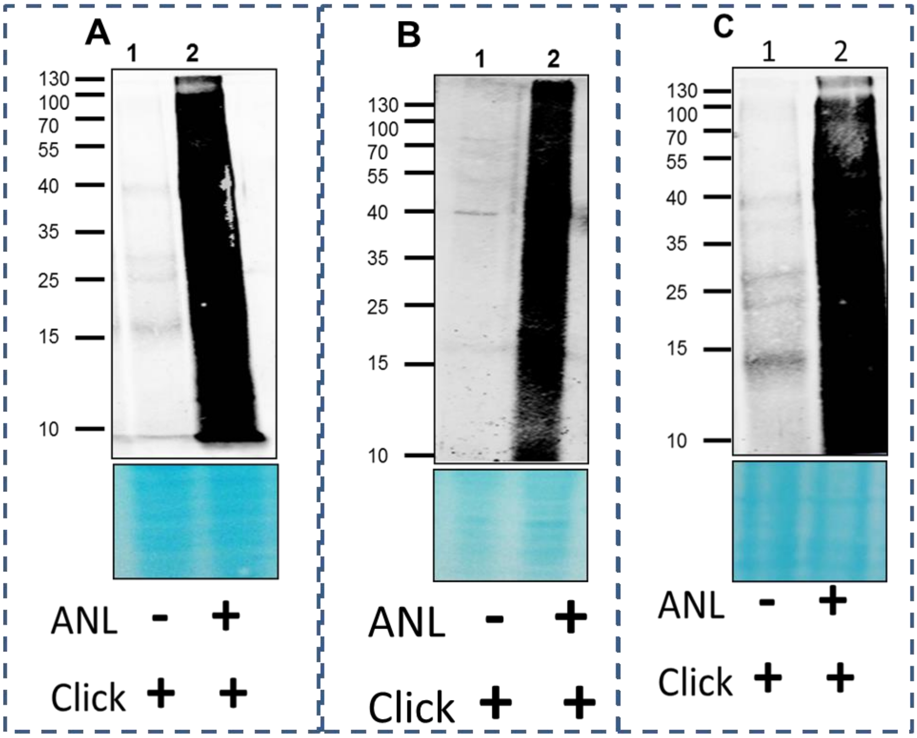

Suppl. Fig-2. Metabolic incorporation of ANL into proteins of H37Rv (A), H37Ra (B) and BND433 (C): Cell were grown in the presence (+) or absence (-) of ANL, and all the cell lysates were treated with biotin-alkyne for covalent incorporation using click reaction. Streptavidin –IR800CW dye signal was captured using Odyssey infra-red scanning system. Stained membrane (bottom) served as a loading control. The signal indicates streptavidin interaction with the covalently captured biotin-alkyne to the ANL incorporated into cellular proteins.

Supplementary Fig. 3

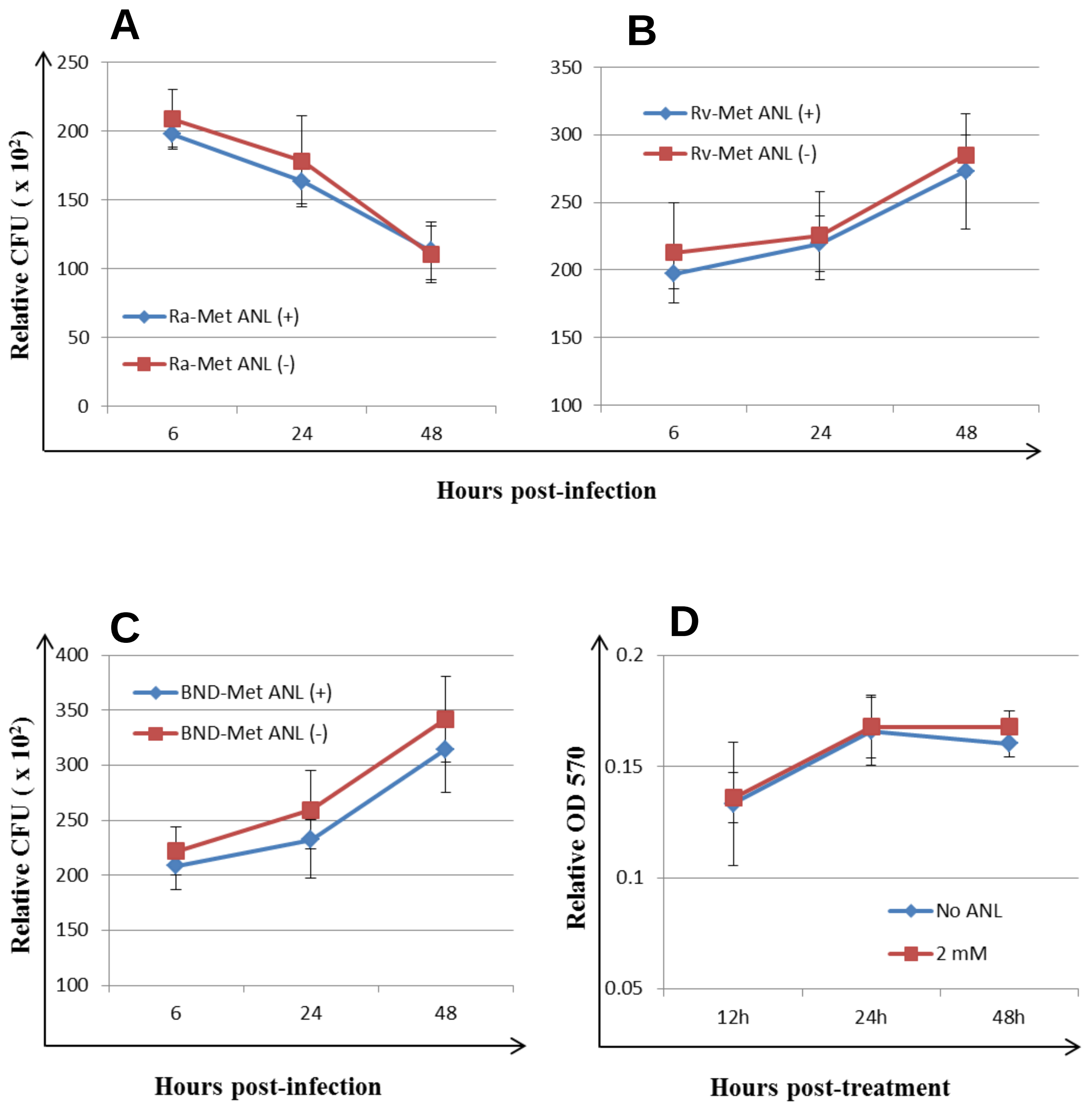

Suppl. Fig-3. ANL toxicity: growth properties of the individual isolates (A-C) and the THP1 host macrophages (D) in presence or absence of ANL. For the mycobacterial isolates CFU values, expressed as a function of viable mycobacterial cells obtained at the indicated times after infection, are shown. Whereas the effect of ANL on the host was examined by MTT assay. (n=3, ±SD)

Supplementary Fig. 4

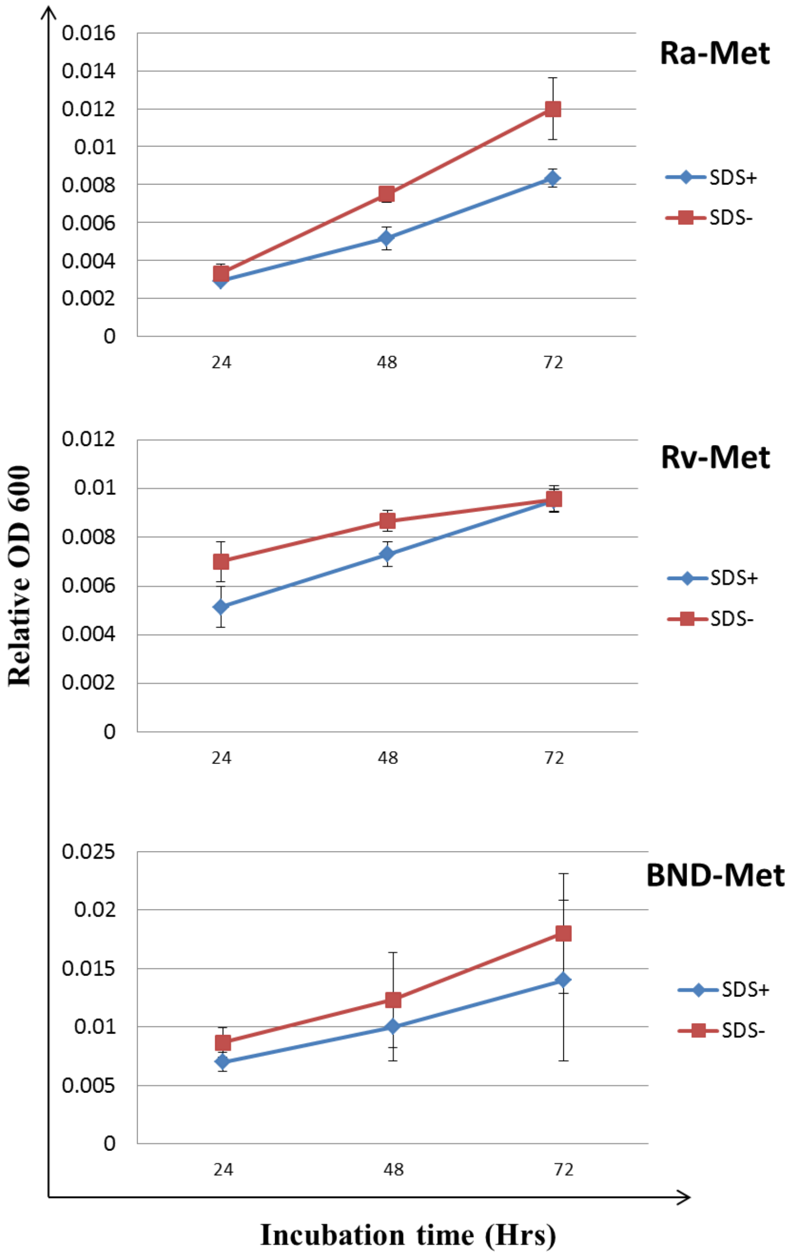

Suppl. Fig-4. Extracellular growth properties of the individual isolates: Mycobacteria were recovered from the infected host by lysis with 1% SDS followed by centrifugation. Recovered bacteria were washed with PBS three times and then inoculated in the middlebrook 7H9 medium. The optical density (OD;600) obtained at the indicated times is shown. Untreated 7H9 broth cultures (SDS-) served as the respective controls for each of the strain under study. (n=3, ±SD)

Supplementary Fig. 5

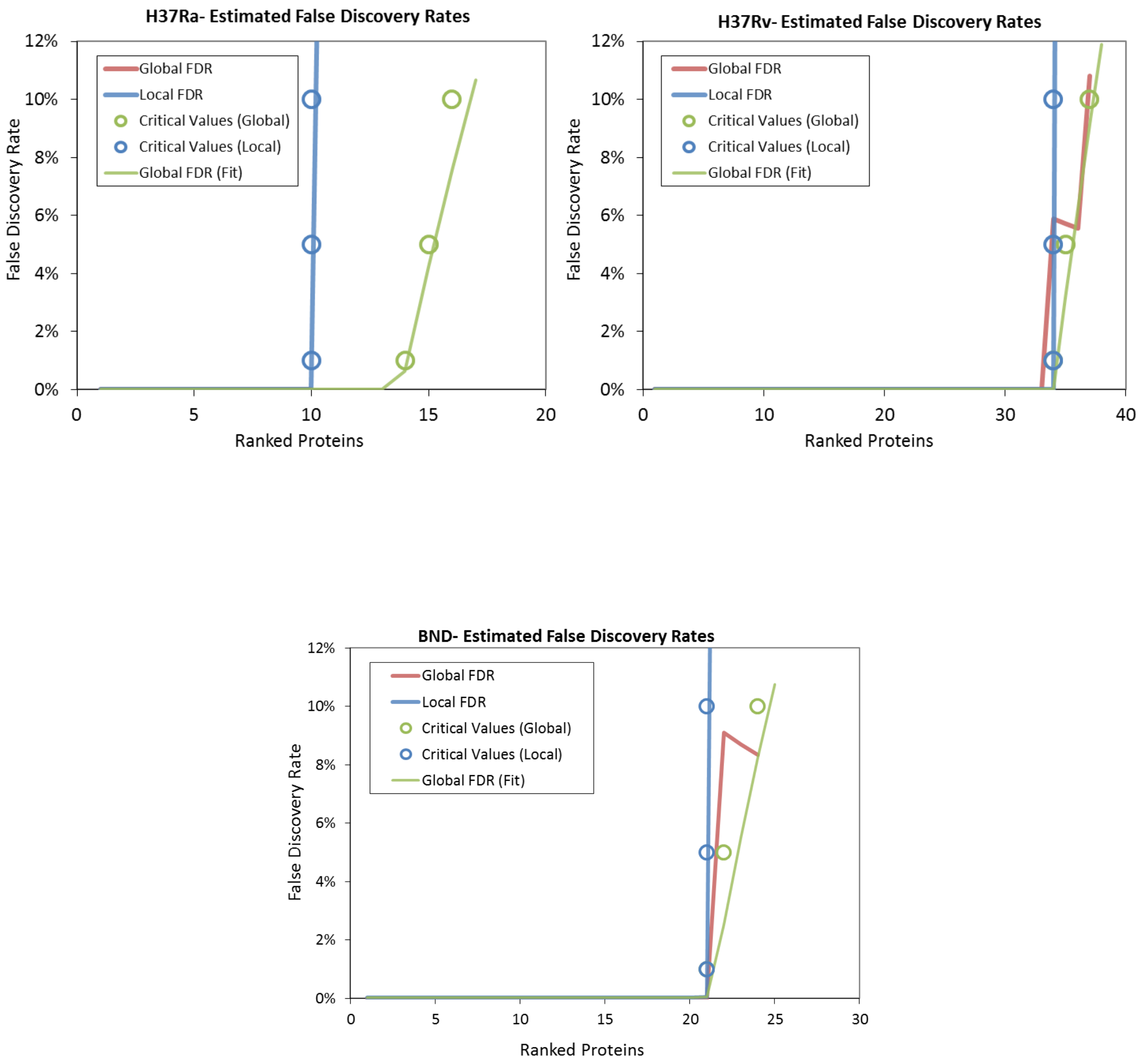

Suppl. Fig-5. Measured False Discovery Rates for MTb Strains: Threshold of 5% accepted Global False discovery rate (G-FDR-fit) proteins criteria were selected for identification of Mtb secreted proteins.

Sequence of Myco-MetRS:

ATGACCCAGGTGGCCAAGAAAATCCTGGTGACCTGCGCCAACCCGTACGCC
AACGGCTCGATCCACCTGGGCCACATGCTGGAACACATCCAGGCCGACGTG
TGGGTGCGCTACCAGCGCATGCGTGGCCACGAGGTGAACTTCATCTGTGCC
GACGACGCCCACGGCACCCCGATCATGCTGAAGGCCCAGCAGCTGGGCAT
CACCCCCGAGCAGATGATCGGCGAGATGTCGCAAGAGCACCAGACCGACT
TCGCCGGTTTCAACATCTCGTACGACAACTACCACTCGACCCACTCGGAGG
AAAACCGCCAGCTGTCGGAGCTGATCTACTCGCGCTTGAAAGAGAACGGC
TTCATCAAGAACCGCACCATCTCGCAGCTGTACGACCCGGAAAAGGGCATG
TTCCTGCCGGACCGCTTCGTGAAGGGCACGTGCCCGAAGTGCAAGTCGCC
GGACCAGTACGGCGACAACTGCGAGGTGTGCGGTGCCACCTACTCGCCGA
CCGAGCTGATCGAGCCGAAGTCGGTGGTGTCGGGTGCCACCCCGGTGATG
CGCGACTCGGAGCACTTCTTCTTCGACCTGCCGTCGTTCTCGGAGATGCTG
CAGGCCTGGACCCGCTCGGGTGCCCTGCAAGAACAGGTGGCCAACAAGAT
GCAAGAGTGGTTCGAGTCGGGCTTGCAGCAGTGGGACATCTCGCGTGATG
CCCCGTACTTCGGCTTCGAGATCCCGAACGCCCCGGGTAAGTACTTCTACGT
GTGGCTGGATGCCCCGATCGGCCTGATGGGCTCGTTCAAGAACCTGTGCGA
CAAGCGTGGCGACTCGGTGTCGTTCGACGAGTACTGGAAGAAGGACTCGA
CCGCCGAGCTGTACCACTTCATCGGCAAGGATATCGTGTACTTCCTGTCGCT
GTTCTGGCCGGCCATGCTGGAAGGCTCGAACTTCCGCAAGCCGTCGAACCT
GTTCGTGCACGGCTACGTGACCGTGAACGGTGCCAAGATGTCGAAGTCGC
GTGGCACCTTCATCAAGGCCTCGACCTGGCTGAACCACTTCGACGCCGACT
CGCTGCGCTACTACTACACCGCCAAGCTGTCGTCGCGCATCGACGACATCG
ACCTGAACTTGGAGGACTTCGTGCAGCGCGTGAACGCCGACATCGTGAAC
AAGGTGGTGAACCTGGCCTCGCGCAACGCCGGTTTCATCAACAAGCGCTTC
GACGGCGTGCTGGCCTCGGAATTGGCCGACCCGCAGCTGTACAAGACCTTC
ACCGATGCCGCCGAGGTGATCGGCGAGGCCTGGGAGTCGCGTGAGTTCGG
TAAGGCCGTGCGCGAGATCATGGCCCTGGCCGATCTGGCCAACCGCTACGT
GGACGAGCAAGCCCCGTGGGTGGTGGCCAAGCAAGAAGGTCGCGACGCC
GACCTGCAGGCCATCTGCTCGATGGGCATCAACCTGTTCCGCGTGCTGATG
ACCTACCTGAAGCCGGTGCTGCCGAAGCTGACCGAGCGTGCCGAGGCCTT
CCTGAACACCGAGCTGACCTGGGACGGCATCCAGCAGCCGCTGCTGGGCC
ACAAGGTGAACCCGTTCAAGGCCCTGTACAACCGCATCGACATGCGCCAG
GTCGAGGCCCTGGTCGAAGCCTCGAAGGAAGAGGTGAAAGCCGCCGCCGC
CCCGGTCACCGGTCCGTTGGCCGATGATCCGATCCAAGAAACCATCACCTT
CGACGACTTCGCCAAGGTGGACCTGCGCGTGGCCCTGATCGAGAACGCCG
AGTTCGTCGAGGGCTCGGACAAGCTGCTGCGCCTGACCCTGGACCTGGGT
GGCGAGAAGCGCAACGTGTTCTCGGGCATCCGCTCGGCCTACCCAGATCCG
CAGGCCTTGATCGGTCGCCACACCATCATGGTGGCCAACCTGGCCCCGCGT
AAGATGCGCTTCGGCATCTCGGAGGGCATGGTGATGGCCGCCGGTCCGGGT
GGCAAGGACATCTTCCTGCTGTCGCCGGATGCCGGTGCCAAACCGGGTCAC
CAGGTGAAGTGA

Note: underlined nucleotides were changed following the Mycobacterial codon usage.
